# Poly(Ionic Liquid) Semi-Interpenetrating Network Multi-Responsive Hydrogels

**DOI:** 10.3390/s16020219

**Published:** 2016-02-06

**Authors:** Alexandru Tudor, Larisa Florea, Simon Gallagher, John Burns, Dermot Diamond

**Affiliations:** Insight Centre for Data Analytics, National Centre for Sensor Research, School of Chemical Sciences, Dublin City University, Dublin, Dublin 9, Ireland; alexandru.tudor2@mail.dcu.ie (A.T.); simon.gallagher24@mail.dcu.ie (S.G.); john.burns9@mail.dcu.ie (J.B.); dermot.diamond@dcu.ie (D.D.)

**Keywords:** smart materials, hydrogels, poly(ionic liquid)s, LCST, polyelectrolyte effect, stimuli-responsive

## Abstract

Herein we describe poly(ionic liquid) hydrogel actuators that are capable of responding to multiple stimuli, namely temperature, ionic strength and white light irradiation. Using two starting materials, a crosslinked poly ionic liquid (PIL) and a linear poly(*N*-isopropylacrylamide-*co*-spiropyran-*co*-acrylic acid), several semi-interpenetrating (sIPN) hydrogels were synthesised. The dimensions of hydrogels discs were measured before and after applying the stimuli, to quantify their response. Samples composed of 100% crosslinked PIL alone showed an average area reduction value of ~53% when the temperature was raised from 20 °C to 70 °C, ~24% when immersed in 1% w/w NaF salt solution and no observable photo-response. In comparison, sIPNs containing 300% w/w linear polymer showed an average area reduction of ~45% when the temperature was raised from 20 °C to 70 °C, ~36% when immersed in 1% NaF w/w salt solution and ~10% after 30 min exposure to white light irradiation, respectively. Moreover, by varying the content of the linear component, fine-control over the photo-, thermo- and salt response, swelling-deswelling rate and mechanical properties of the resulting sIPN was achieved.

## 1. Introduction

Stimuli-responsive polymers are defined as polymers that exhibit a conformation change in a reliable, reproducible and useful manner when exposed to a variation in their external environment [1,2]. Many types of stimuli can be employed, including temperature, pressure, magnetic and electric field, lighting conditions, pH and the presence of other chemical species in their local medium [1,3,4,5,6,7,8,9,10,11,12,13,14,15,16,17,18,19,20]. Hydrogels comprise a particularly interesting class of stimuli-responsive materials due to their biomimetic nature [6]. These materials are crosslinked polymer networks that absorb large quantities of water and, when under the influence of specific stimuli, change their water absorption properties, which in turn translate into a hydrogel volume variation [7,14]. Extensively studied examples of such materials include *N*-isopropylacrylamide (NiPAAm) hydrogels, which possess a well-documented thermally induced shrinking behaviour associated with a lower critical solution temperature (LCST) at *ca.* 32–35 °C [4,5,9,12,13,14,15,21,22,23,24]. This behaviour is a result of a thermodynamically driven equilibrium shift from the strongly hydrated form defined by solution-polymer interactions to a much more compact form dominated by polymer-polymer interactions. Copolymerization of NiPAAm with other stimuli-responsive units, such as photochromic dyes, offers the possibility of creating hydrogels that respond to additional stimuli such as light [4,12,25].

Ziołkowski *et al.* recently described the synthesis and characterization of a photo/thermo-responsive hydrogel material consisting of NiPAAm-*co*-spiropyran-*co*-acrylic acid (p(NiPAAm-BSP-AA)) copolymer [4]. The additional photo-response is conferred by the presence of the spiropyran photochromic monomer that reversibly changes its conformation from a hydrophilic protonated merocyanine form (Mc-H^+^) to a hydrophobic spiropyran form (SP) under different illumination conditions. When immersed in DI water, the acrylic acid comonomer dissociates and the spiropyran is protonated to form the Mc-H^+^ form. In this conformation, the molecule is more hydrophilic compared to the closed SP form, triggering hydrogel expansion. Upon white light irradiation, the reverse process happens, and the Mc-H^+^ is converted back to the closed SP form. Thus, based on this photo-induced transition of the polymer from a more hydrophilic to a more hydrophobic conformation, water is expelled from the hydrogel matrix. This effect caused some of the hydrogels to shrink by ~49%, compared to their maximum shrinking capability, after a 20 min exposure to white-light. The gels subsequently revert back to ~97% of their original size after 1 h in the dark. 

A new class of polymeric materials that feature LCST behaviour are a series of phosphonium-based poly(ionic liquid)s (PILs) [3,8,10,11,15,26,27,28]. These materials represent a subgroup of ionic liquids that include polymerizable groups in the cation, the anion or both, respectively. The PILs inherit the properties of ionic liquids, such as very low vapour pressure, thermal stability and high ionic conductivity [29,30,31], while they can also be polymerized, thus can be used in the production of membranes, hydrogels, coatings and films. A recent paper by Gallagher *et al.* discusses the improved thermal response of a semi-interpenetrating network (sIPN) synthesized by polymerizing a phosphonium PIL in the presence of linear pNiPAAm chains [15]. The addition of linear pNiPAAm chains to the crosslinked PIL matrix improved the temperature-induced shrinking at 75 °C of the PIL hydrogels by ~13% when compared to the crosslinked PIL without any linear pNiPAAm chains.

In this context, the focus of the following study was the synthesis of multi-responsive sIPN hydrogels using a tributylhexyl phosphonium 3-sulfopropyl acrylate PIL crosslinked matrix and controlled amounts of linear p(NiPAAm-BSP-AA) copolymer chains. The shrinking properties of these hydrogels under the influence of temperature, white light irradiation and salt solutions were described. Further characterization of the linear copolymer was done using Differential Scanning Calorimetry (DSC) and UV-Vis spectroscopy, while the curing properties of the sIPNs were analysed using rheometry.

## 2. Experimental Section

### 2.1. General Information

*N*-isopropylacrylamide 97% (NiPAAm) (100 ppm MEHQ as inhibitor), phenyl-bis(2,4,6-trimethylbenzoyl) phosphine oxide 97% (PBPO), potassium 3-sulfopropyl acrylate (KSPA), polypropylene glycol diacrylate (M_w_ ~800, 100 ppm MEHQ and 100 ppm BHT as inhibitors) (PPG800), sodium fluoride >99%, sodium chloride >99%, sodium iodide >99% were bought from Sigma Aldrich^®^ (Arklow, Ireland) and used as received, sodium bromide >99% and HPLC grade acetonitrile (ACN) were also bought from Sigma Aldrich^®^ (St. Louis, MO, USA) and used as received. The tributylhexyl phosphonium chloride (P_4446_Cl) was kindly donated by Cytec^®^ Industries (Niagara Falls, NY, USA). Trimethyl-6-hydroxyspiro-(2H-1-benzopyran-2,2′-indoline) acrylate (BSP) was synthesized according to a previous procedure [4]. Deionized water (18.2 MΩ·cm^−1^) (DI water) was made using a Milli-Q Water Purification System (Merck Millipore, Darmstadt, Germany). ^1^H-NMR spectra were recorded at 400 MHz on a Bruker Advance Ultrashield NMR spectrometer (Coventry, UK). UV-Vis spectra were recorded on a Lambda 900 spectrometer (Perkin-Elmer, Waltham, MA, USA). Temperature control was achieved via a Perkin Elmer PTP-1 Peltier Temperature Programmer. (DSC) was performed using a Perkin-Elmer Pyris 1 DSC, which was calibrated using an indium standard with a melting point of 156.6 °C. Rheometry experiments were performed using a MCR301 rheometer (Anton-Paar, Graz, Austria).

### 2.2. Synthesis of Tributylhexyl Phosphonium 3-Sulfopropyl Acrylate Ionic Liquid Monomer

**Scheme 1:**
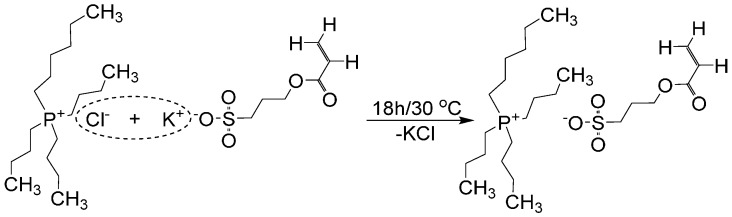
The PSPA synthesis reaction.

The synthesis of tributylhexyl phosphonium 3-sulfopropyl acrylate (PSPA) was carried out by dissolving P_4446_Cl (5 g) and KSPA (4.628 g, 1.3 molar equivalents) in deionized water (25 mL), followed by stirring at 30 °C for 18 h (Scheme 1). The reaction product was extracted three times with dichloromethane (DCM, 25 mL). The resulting organic phase was washed with deionized water to extract any unreacted products. Following this final separation, anhydrous MgSO_4_ was added and the resulting solution was filtrated. The PSPA organic solution was concentrated using a rotary evaporator, followed by extracting the final solvent traces using a high vacuum pump (0.5 mBar). The yield of the synthesis and purification method was around ~65%. PSPA—^1^H-NMR, δ_H_ (400 MHz, CDCl_3_): 0.84–0.95 (m, 12H, CH_3_), 1.26–1.29 (m, 4H, CH_2_), 1.47–1.50 (m, 16H, CH_2_), 2.14–2.32 (m, 10H, CH_2_), 2.84–2.87 (t, 2H, CH_2_), 4.22–4.25 (t, 2H, CH_2_), 5.74–5.77 (dd, 1H, CH), 6.01–6.08 (m, 1H, CH), 6.31–6.36 (dd, 1H, CH) ppm.

### 2.3. Synthesis of Linear Poly(N-Isopropylacrylamide-co-Spiropyran-co-Acrylic Acid) p(NiPAAm-BSP-AA) Copolymer

For the synthesis of linear p(NiPAAm-BSP-AA) copolymer, NiPAAm (1.13 g) was dissolved in THF (5 mL) along with 1 mol% eq. of PBPO, 1 mol% eq. BSP and 5 mol% eq. acrylic acid. The mixture was stirred until all of the components were completely dissolved. The resulting solution was then photopolymerized for 20 min with the help of a LMI-6000 Fiber-Lite white light source (~200 kLux, Dolan Jenner Industries, Boxborough, MA, USA). Finally, after the polymerization was finished, the resulting dissolved polymer was precipitated in cold diethyl ether. Afterwards, the precipitate was vacuum-filtered and dried in a vacuum oven at 40 °C for one hour to yield the p(NiPAAm-BSP-AA) copolymer. 

### 2.4. Synthesis of the Semi-Interpenetrating Network Hydrogels

**Figure 1:**
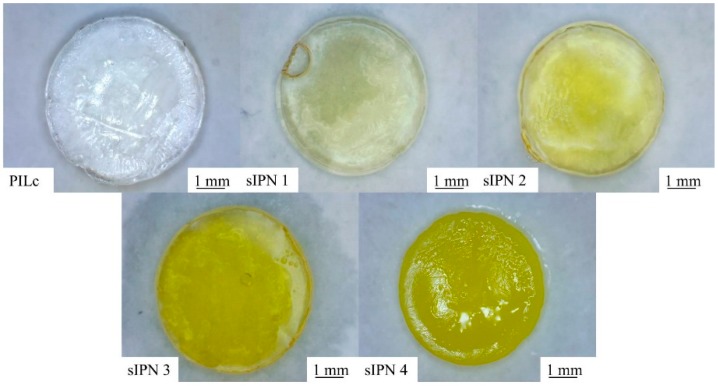
The photopolymerized hydrogels after swelling in DI water at 20 °C for 24 h.

The semi-interpenetrating networks (sIPNs) were synthesized by adding the required amount of PSPA to a series of linear p(NiPAAm-BSP-AA) copolymer solutions in 1:1 w/w mixture of ACN and DI water, together with 2 mol % eq. PBPO and 5 mol % eq. PPG800 with regard to PSPA (Table 1). The amount of linear copolymer was calculated so that the molar ratio between the PSPA and the NiPAAm co-monomer increases from 1:1 in sIPN 1 to 1:4 in sIPN 4 (Figure 1). This was done to provide some insights into how the increasing amounts of linear p(NiPAAm-BSP-AA) affects the thermo- and photo- induced shrinking capabilities of the resulting sIPN hydrogel.

**Table 1:** Monomeric mixture compositions for the synthesized hydrogels.

	PILc	sIPN 1	sIPN 2	sIPN 3	sIPN 4
**P_4,4,4,6_-SPA (g)**	0.1935	0.1935	0.1935	0.1935	0.1935
**Linear polymer (g)**	**0**	**0.0453**	**0.0905**	**0.1308**	**0.1810**
**P_4,4,4,6_-SPA:NiPAAm (molar ratio)**	**1:0**	**1:1**	**1:2**	**1:3**	**1:4**
**PPG 800 (g)**	0.0160	0.0160	0.0160	0.0160	0.0160
**PBPO (g)**	0.0034	0.0034	0.0034	0.0034	0.0034
**ACN:H_2_O (g)**	0.1935	0.1935	0.1935	0.1935	0.1935

The monomer solutions were mechanically shaken until all of the components were dissolved. Following this, volumes from each mixture were transferred into a polydimethylsiloxane mould with wells of 3 mm diameter and 1 mm depth, and photopolymerized in the wells using the LMI-6000 Fiber-Lite white light source (~200 kLux) for 20 min. After the polymerization was complete, the gels were hydrated in DI water until they were fully swollen.

### 2.5. Thermal Behaviour of Linear p(NiPAAm-SPA-AA) Copolymer

UV-Vis spectrometry was used to determine the presence of the lower critical solution temperature (LCST) in the linear p(NiPAAm-BSP-AA) copolymer. This was achieved by making a 0.1% w/w linear copolymer solution in DI water, and measuring its absorbance at 2 °C temperature steps between 20 °C and 36 °C. The temperature was held constant for 5 min at each temperature step to enable the dynamics of the temperature effect on the polymer to be observed. Two sets of data were gathered, one for the solution kept in the dark and one for the solution after it was irradiated with white light. To confirm the LCST determined using UV-Vis spectrometry, differential scanning calorimetry analysis was employed. The sample, 14.3 mg of 2.5% w/w linear copolymer solution in DI water, was analysed using the following thermal program: heating from 14 °C to 70 °C at a heating rate of 10 °C/min followed by cooling from 70 °C to 14 °C at a cooling rate of 10 °C/min. This process was repeated three times to ensure that the results were reproducible.

### 2.6. White-Light Curing Studies of the sIPN Hydrogels Using Rheometry

Rheometry was used to analyse the photo-induced curing behaviour of PILc and sIPN mixtures described in Table 1. The analysis was performed by fitting the rheometer with the PP15 parallel plate tool and using a strain of 0.1% at a frequency of 1 Hz. Data points were collected every 5 s for a total of 480 s. The rheometer was fitted with a glass slide base to facilitate the photo-polymerization of the monomer mixtures. The white-light source was mounted to provide a ~200 kLux illuminance exposure on the monomer mixture. All measurements were taken at a constant temperature of 25 °C.

### 2.7. Measurement of Stimuli-Induced Shrinking

Quantifying the stimuli-induced shrinking of PILc and sIPN hydrogels was achieved by taking area measurements before and after applying the stimulus. Sample images were taken with a GE-5 digital microscope (Aigo, Beijing, China) fitted with a 60× lens and using the Aigo ScopeImage 9.0 imaging software. The resulting images were analysed using ImageJ image analysis software. For each stimulus measurement three gels were used. The %shrinking of each hydrogel was calculated using the formula:

(1) $$\%\text{shrinking}=100-\left(A_{f}/A_{i}\cdot 100\right)$$

where A_f_ is area of the hydrogel after applying a stimulus and A_i_ is the initial area of the hydrogel. 

The stimuli investigated were: white-light irradiation, ionic strength and temperature. For the white-light induced shrinking, the hydrogels were placed in poly(methyl methacrylate) (PMMA) wells filled with DI water and covered with a glass slide, to prevent evaporation, and irradiated with white light (~200 kLux) for 30 min. In the case of the ionic strength induced shrinking, the gels were swollen in DI water and transferred to 1% w/w solutions of NaF, NaCl, NaBr and NaI, respectively, and left until equilibrium was reached. Temperature measurements were performed using an Anton Paar MCR 301 Rheometer Peltier holder fitted with an aluminium plate. The gels were placed on the plate and covered with either DI water or 0.5% w/w NaCl solution. A glass plate cover was used to avoid evaporation. The temperature program used covered a temperature interval between 20 °C and 70 °C with a heating step of 5 °C. Between each step, the temperature was kept constant for a period of 5 min to ensure the gels reached equilibrium, after which an image was taken for subsequent analysis.

## 3. Results and Discussion

### 3.1. White Light and Temperature Response of the Linear p(NiPAAm-BSP-AA) Copolymer Solutions

**Scheme 2:**
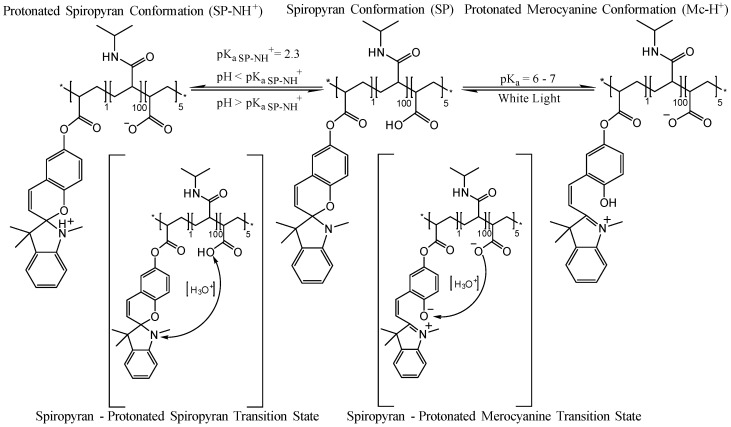
Scheme showing the equilibrium of p(NiPAAm-BSP-AA) copolymer in DI water under different illumination conditions.

When p(NiPAAm-BSP-AA) linear copolymer is dissolved in DI water or in aqueous solutions of pH > 4, the following equilibrium takes place (Scheme 2): in pH solutions of pH > 4 (pK_a_ acrylic acid = 4.2 [4]), the acrylic acid comonomer dissociates and in the transition state, it protonates the merocyanine (Mc) isomer of the spiropyran, forming the yellow-coloured protonated merocyanine (Mc-H^+^, Scheme 2). When the solution is irradiated with white-light the equilibrium shifts towards the closed colourless spiropyran isomer (Scheme 2). The Mc-H^+^ form is more hydrophilic, while the closed spiropyran form is more hydrophobic [4,12]. This equilibrium shift has an important impact on the hydrophilic/hydrophobic character of the dissolved polymer, triggering a bulk conversion of the polymer into a more hydrophobic conformation. Poly(NiPAAm) is a thermo-responsive polymer, meaning that it exhibits lower critical solution temperature (LCST) behaviour, which in the case of poly(NiPAAm) is around 32 °C [5,12,21]. The mechanism behind this behaviour is related to how the solubility equilibrium between the linear polymer chains and the hydrating water molecules changes due to an increase in temperature. Below the LCST, the hydrophilic amide part of NiPAAm forms hydrogen bonds with the water molecules, and the polymer adopts a swollen, strongly hydrated extended coil conformation. In contrast, above the LCST, the polymer-solvent hydrogen bonds weaken to the extent that the hydrophobic interactions between the polymer chains become dominant. This makes the polymer adopt a more compact globular form, which precipitates out of the solution [21]. By co-polymerising additional monomeric units in the poly(NiPAAm), like BSP in this instance, this temperature-response can be converted into a photo-response. The creation of hydrophobic units (spiropyran conformation) inside the polymer matrix through irradiation with white light causes a cascade effect, inducing the precipitation of the polymer chain from the hydrated form. As a consequence, an expulsion of water takes place when the equilibrium is shifted by white light irradiation, and the polymer shrinks.

The determination of the LCST of p(NiPAAm-BSP-AA) is a necessary part of this study, because it enables us to gain an insight into how the additional co-monomers influence the thermo-responsive properties of the linear copolymer [32]. The LCST of the copolymer was determined both qualitatively by UV-Vis spectroscopy and quantitatively by DSC. The UV-Vis LCST analysis was made using a 0.1% w/w copolymer solution in DI water. This allowed the LCST to be determined qualitatively by observing the temperature-dependent absorbance increase at 700 nm. At this wavelength there is no interference from any other peaks present in the absorbance spectrum of the copolymer solution [12]. This means that the increase in absorbance is solely caused by precipitation of linear copolymer in solution. Following the qualitative analysis of the LCST, DSC was chosen to generate quantitative data. During the LCST event there is a change in the hydration energy of the polymer, which appears as an endothermic transition on the DSC curve [5].

The UV-Vis analysis of the non-irradiated sample shows an absorption peak centred at 422 nm, which corresponds to the presence of the protonated merocyanine (Mc-H^+^) conformation in the copolymer solution [12]. This is the dominant species of the SP/Mc/Mc-H^+^ system under these conditions, and it arises due to the dissociation of the AA comonomer and protonation of the MC form to Mc-H^+^ (pK_a_ = 6–7 [12]). This process happens spontaneously when the p(NiPAAm-BSP-AA) copolymer is hydrated in DI water in the dark [4]. The N-protonated SP form (SP-NH^+^, pK_a_ = 2.3 [33]) is formed in a competing side equilibrium and the band with the maximum centre around 316 nm can be assigned to this form [33]. From 20 °C to 28 °C the absorption peak at 422 nm continuously rises, because the acrylic acid dissociation equilibrium is shifted towards release of free protons [34], increasing the amount of Mc-H^+^ present in the linear copolymer. Between 30 °C and 36 °C the absorbance at 422 nm continues to rise, with a concomitant red shift towards 436 nm, which is associated with the formation of Mc-H^+^ J-aggregates [35]. Simultaneously, the absorbance at 700 nm starts growing, indicating that precipitation is happening. 

**Figure 2:**
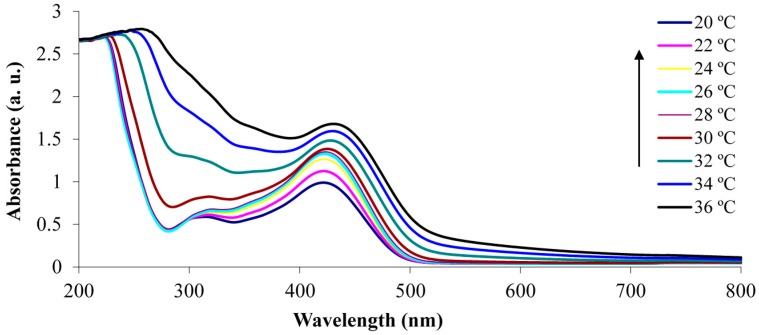
UV-Vis absorbance spectra of the non-irradiated 0.1% w/w p(NiPAAm-BSP-AA) linear copolymer solution in DI water.

**Figure 3:**
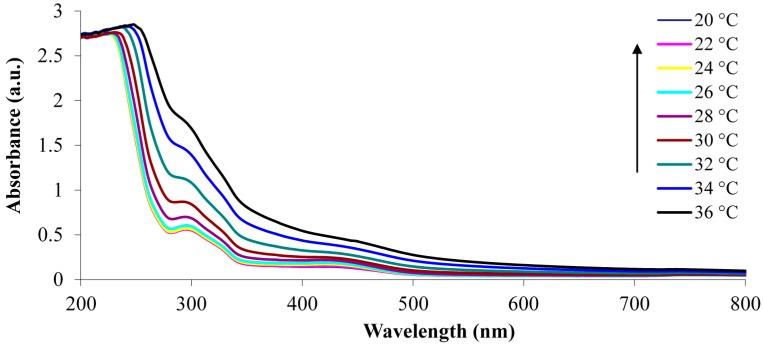
UV-Vis absorbance spectra of the white light irradiated 0.1% w/w p(NiPAAm-BSP-AA) linear copolymer solution in DI water.

The same solution was then irradiated with white light (~250 kLux) for 5 min before the absorbance spectrum was recorded at each temperature (Figure 2). After the solution was irradiated, its colour changed from bright yellow to colourless, due to conversion of Mc-H^+^ to the closed SP form (Figure 3). This is confirmed by the decrease of the peak at 422 nm. The absorbance peak centred at 294 nm shown in Figure 3 due to the presence of the SP isomer, while the shoulder at ~316 nm probably indicates the presence of the SP-NH^+^ form [33].

**Figure 4:**
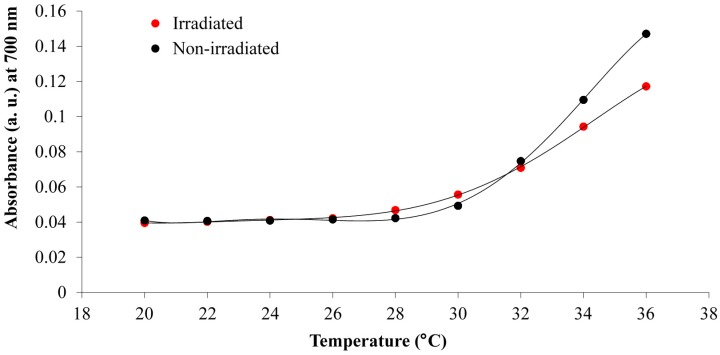
Comparison of the absorbance values at 700 nm between the non-irradiated and white light irradiated 0.1% w/w p(NiPAAm-BSP-AA) linear copolymer solutions at different temperatures.

In Figure 4 the rise in absorbance at 700 nm between the non-irradiated and white light irradiated samples is compared. The absorbance at 700 nm for the white light irradiated solution begins to increase at a lower temperature (~28 °C) compared to the non-irradiated solution (~30 °C), which is in accordance with results reported previously by Sumaru *et al*. [12]. This phenomenon occurs because of the hydrophobicity increase of the polymer chains when irradiated with white light. The BSP form is more hydrophobic compared to Mc-H^+^, thus promoting the precipitation of the linear p(NiPAAm-BSP-AA) chains at a lower temperature [32]. After 32 °C, the non-irradiated solution absorbance crosses the irradiated solution absorbance, which can be explained by additional formation of J aggregates between the Mc-H^+^ molecules in the linear p(NiPAAm-BSP-AA) copolymer chains which facilitates chain precipitation.

**Figure 5:**
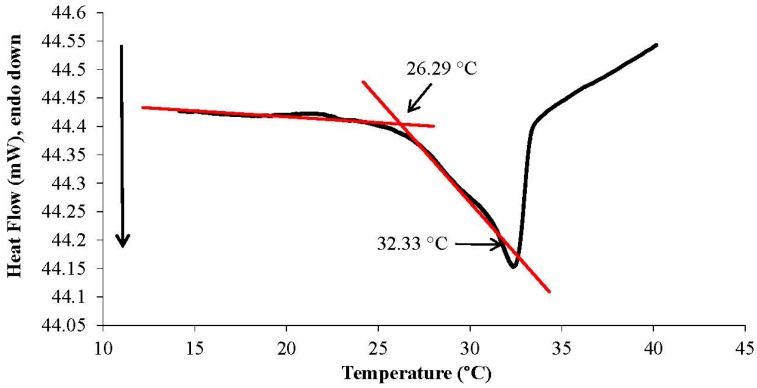
DSC curve excerpt showing the LCST transition of a 2.5% w/w p(NiPAAm-BSP-AA) linear copolymer solution. The full DSC curve follows a heating program from 14 °C to 70 °C at a heating rate of 10 °C/min.

Following the qualitative analysis of the LCST, DSC was used to obtain quantitative data. The DSC analysis of the 2.5% linear copolymer w/w solution determined an onset temperature at 26.29 °C and a maximum peak temperature at 32.33 °C (Figure 5). The onset temperature corresponds to the temperature at which the solution starts to undergo the precipitation process, while the peak temperature corresponds to the temperature at which the system completely precipitates (*i.e.*, the LCST). These results correlate with the UV-Vis measurements, showing in both cases that the linear copolymer starts to precipitate between 26–28 °C and continues up to 36 °C. Moreover, the DSC curve gives a very close LCST value to the 32–35 °C value quoted in literature for poly(*N*-isopropylacrylamide), the main component of the p(NiPAAm-BSP-AA) copolymer [5]. 

### 3.2. Photo-Induced Curing Studies and Mechanical Properties of the Hydrogels

**Figure 6:**
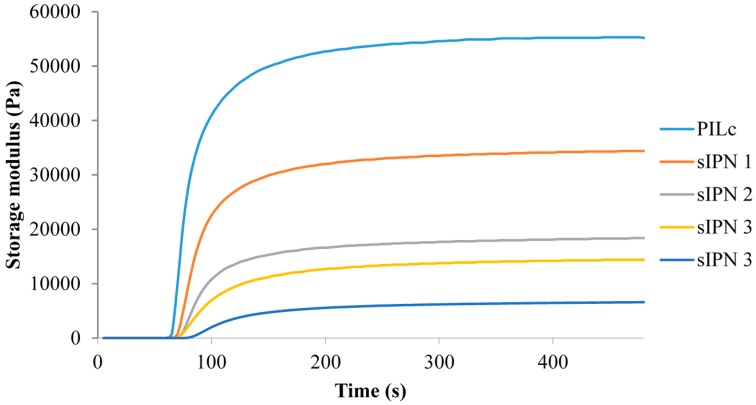
Time-dependent variation of the storage modulus of the monomer cocktail during polymerization. The white light is turned on at *t* = 60 s.

To determine the curing and mechanical properties of the hydrogels, samples of PILc, sIPN 1, sIPN 2, sIPN 3 and sIPN 4 hydrogels were synthesized in triplicate, according to the cocktail compositions given in Table 1. The storage modulus curves in Figure 6 show that each mixture features a sharp increase in the storage modulus value after being exposed to white light at *t* = 60 s. This is due to the rapid initiation and propagation of the photo-polymerization reaction. Following this, the storage modulus begins to plateau at approximately the same time for every monomer mixture used, indicating that the storage modulus reached ~95% of its maximum value after *ca.* 120 s exposure to white-light. 

From the same data, additional observations can be made about the mechanical properties of the resulting IPN hydrogels and the monomer cocktail compositions. By increasing the amount of linear copolymer a decrease in the storage modulus of the hydrogels can be seen (Figure 6). For example, when increasing the amount of linear copolymer from 1:1 (P_4,4,4,6_-SPA:NiPAAm, molar ratio) in sIPN 1 to 1:2 (P_4,4,4,6_-SPA:NiPAAm) for sIPN 2, the storage modulus decreases from 31.4 kPa to 16.3 kPa. The lower storage modulus indicates that the hydrogels are more brittle and prone to breaking when handled. Tanδ represents the ratio between the loss and storage modulus of a fluid and is a measure of the viscoelastic behaviour of a material [24]. If the value is below 1, it indicates that the sample has a more elastic-like behaviour, while if the value is greater than 1, then the sample has a more viscous-like behaviour [24].

Based on the tanδ values calculated for the PILc and sIPN hydrogels, all of the hydrogels have an elastic-like behaviour (Table 2). The increasing value of tanδ with increasing amount of the linear p(NiPAAm-BSP-AA) is confirmed by the increased tendency of the hydrogels to become tackier after polymerization, thus making them harder to manipulate.

**Table 2:** Loss and storage modulus of the hydrogels at 25 °C after 180 s of white light irradiation.

Hydrogel	NiPAAm (molar %–to PSPA)	Loss Modulus (Pa)	Storage Modulus (Pa)	Tanδ
**PILc**	0	76	51,800	**0.0015**
**sIPN 1**	100	85.7	31,400	**0.0027**
**sIPN 2**	200	650	16,300	**0.0400**
**sIPN 3**	300	617	12,200	**0.0506**
**sIPN 4**	400	832	5310	**0.1567**

**Figure 7:**
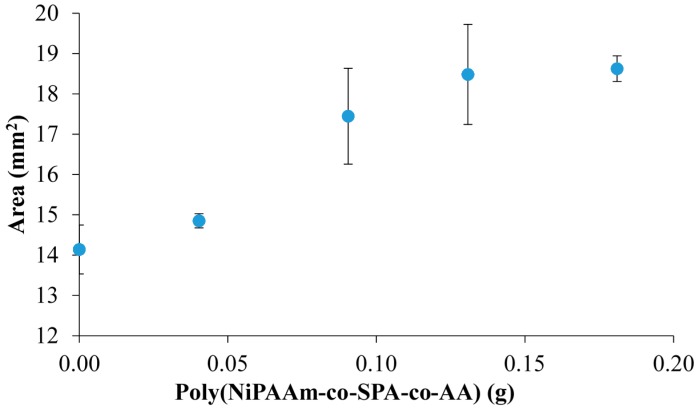
The effect of increasing the mass of linear p(NiPAAm-BSP-AA) copolymer on the fully hydrated hydrogel area (error bars are standard deviations for n = 3 replicate measurements).

Another effect caused by the increasing quantity of linear copolymer in the PILc matrix is an observable increase in swelling area when the gels are left to hydrate in DI water (Figure 7, Table 3). The addition of linear p(NiPAAm-BSP-AA) increases the hydrophilic character of the hydrogels, because the co-monomers are hydrophilic molecules at room temperature. 

**Table 3:** The effect of increasing amount of linear p(NiPAAm-BSP-AA) copolymer on the hydration properties of the hydrogels.

Hydrogel	NiPAAm (molar %–to PSPA)	Hydrated Area (mm^2^)	Standard Deviation (n = 3)	Hydrated Area Increase (%)	RSD (%) (n = 3)
**PILc**	0	14.140	0.606	-	4.286
**sIPN 1**	100	14.851	0.177	5.030	1.193
**sIPN 2**	200	17.445	1.191	23.375	6.825
**sIPN 3**	300	18.482	1.242	30.708	6.717
**sIPN 4**	400	18.625	0.317	31.715	1.703

The maximum increase in hydrated area (31.72% ± 1.70%) is obtained for sIPN 4 relative to the PILc in the absence of copolymer. To calculate this, the following formula was used:

(2) $$Hydrated\;Area\;Increase\;\left(\%\right)=100-\;\left(\frac{sIPN_{x}\;Hydrated\;Area}{PILc\;Hydrated\;Area}*100\right)$$

where sIPN_x_ is sIPN 1, sIPN 2, sIPN 3 and sIPN 4, respectively. 

### 3.3. White Light Induced Shrinking

**Figure 8:**
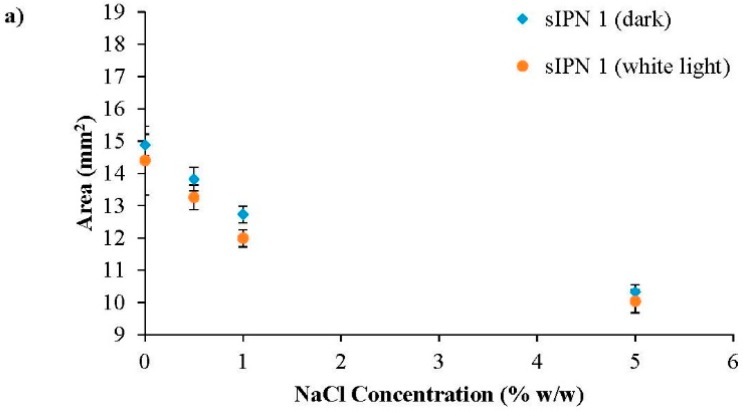

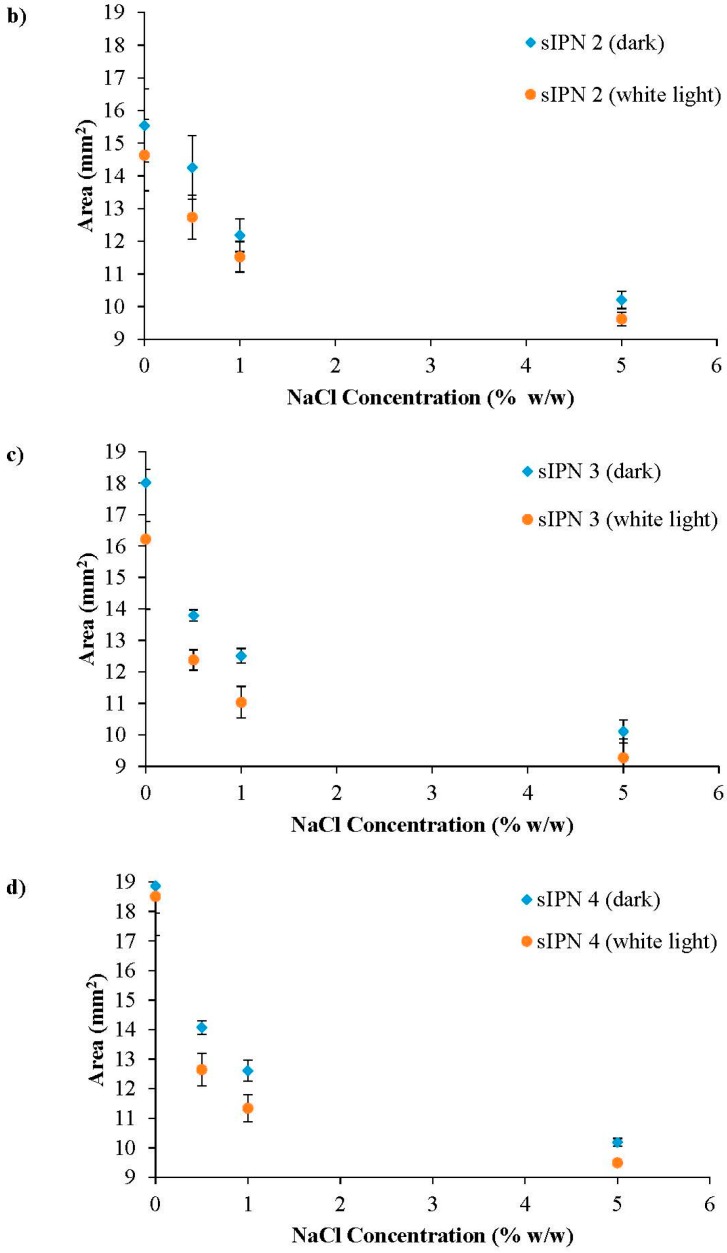
White-light and ionic strength induced shrinking of the hydrogels. (**a**) sIPN 1; (**b**) sIPN 2; (**c**) sIPN 3; (**d**) sIPN 4.

**Figure 9:**
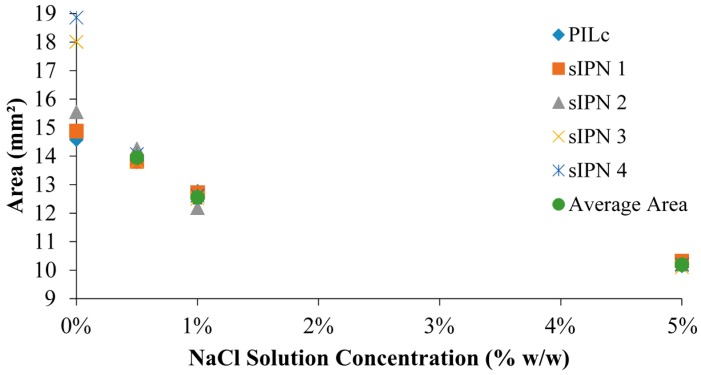
Area of the hydrogels in DI water and NaCl solutions of different concentrations: 0.5%, 1% and 5%, respectively, in the absence of light.

The inclusion of the white-light sensitive linear copolymer, p(NiPAAm-BSP-AA), in the sIPN matrix makes it possible to use the Mc-H^+^->SP photo-induced conformation change to influence the degree of swelling of the hydrogel [4,12]. The photo-induced shrinking of the different sIPN hydrogels was tested in different hydration media: DI water, 0.5% w/w NaCl, 1% w/w NaCl and 5% w/w NaCl, respectively. The effect of salt solutions on NiPAAm-based hydrogels has already been studied, and it was found that at 25 °C and at salt concentrations higher than 4% w/w NaCl, the dissolved linear pNiPAAm polymer precipitates out of solution [22]. This is due to the Na^+^ and Cl^−^ ions competing for water with the linear p(NiPAAm), enhancing the tendency of p(NiPAAm) to form polymer-polymer interactions and to more readily precipitate as the globular form. This is the same phenomenon that appears when salting-out proteins from a solution [7,36]. A similar effect of the salt concentration is expected with the p(NiPAAm-BSP-AA) chains when the sIPN is hydrated in aqueous salt solutions. In Figure 8a–d, the area of sIPN 1, sIPN 2, sIPN 3 and sIPN 4 is plotted against salt concentration. For each hydrogel two area values were plotted for every salt concentration, representing the hydrogel’s area before and after being irradiated with white light for 30 min at ~200 kLux. The largest area decreases occur with sIPN 2 and sIPN 3 when immersed 0.5% w/w NaCl solutions. For sIPN 2 the decrease in area is 10.77% ± 5.24% (n = 3), while for sIPN 3 the decrease is 10.26% ± 2.59% (n = 3). The results indicate that for each hydrogel, the white-light induced shrinking remains roughly constant until the salt concentration exceeds 1% w/w NaCl. At higher salt concentrations, the hydrogels primarily shrink in size due to the interactions that start taking place between the dissolved salt ions and the charged polymer chains, while the white light irradiation shows reduced influence. This shrinking behaviour is known as the polyelectrolyte effect [7,26]. For each hydrogel composition, the shrinking profile is very similar as the salt concentration is increased (13.95 ± 0.21 mm² in 0.5% w/w NaCl, 12.56 ± 0.23 mm² in 1% w/w NaCl and 10.00 ± 0.09 mm² in 5% NaCl, respectively) (Figure 9). 

### 3.4. Ionic Radius Dependent Shrinking 

**Figure 10:**
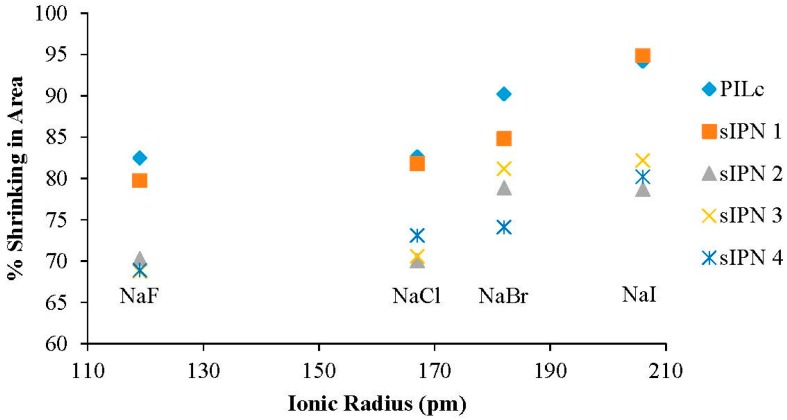
The shrinking effect of 1% w/w NaF, NaCl, NaBr and NaI solutions on the area of the hydrogels compared to DI water.

To better understand the effect of dissolved ions in the hydrating solution on the hydration properties of the hydrogels, a series of 1% w/w solutions were made using NaF, NaCl, NaBr and NaI, respectively. In Figure 10, the area of each hydrogel was plotted against the ionic radius of the anions present in the hydrating solutions. The shrinking% was calculated, using Equation (1), taking into account that the fully hydrated area was considered the area of the hydrogels swollen in DI water. By plotting the shrinking effect against the ionic radius of the anions, the influence of volume charge density can be explained. Volume charge density is a measure of electric charge per unit of volume. The results indicate that F^−^ induces the maximum area shrinking for every hydrogel, while I^−^ has the least influence (Table 4). The reason behind this shrinking effect is two-fold: the dissolved salts have a shrinking effect on the PIL matrix due to the polyelectrolyte effect [7], while also having the same effect on the linear p(NiPAAm-BSP-AA) chains [22,32,36]. The shrinking effect occurs because of an electrostatic screening that appears between the charged PIL chains and the ions present in the solution that affects the way water is distributed around the polymer chains and salt ions, thus directly contributing to the water intake of the hydrogels [37]. The fluoride anion has the highest volume charge density and therefore has the strongest screening effect, making the hydrogels shrink the most. In contrast, iodide has the lowest volume charge density, making the hydrogels shrink the least. 

**Table 4:** Salt-induced shrinking of the hydrogels in area % compared to DI water.

	A^−^ Ionic Radius (pm)	PILc (%)	sIPN 1 (%)	sIPN 2 (%)	sIPN 3 (%)	sIPN 4 (%)
**NaF**	119	82.491	79.760	70.316	68.742	68.912
**NaCl**	167	82.621	81.791	70.058	70.582	73.098
**NaBr**	182	90.246	84.833	78.894	81.213	74.110
**NaI**	206	94.196	94.898	78.703	82.195	80.228

### 3.5. Temperature Induced Shrinking

In the case of crosslinked PILs, the LCST is a temperature interval, not an exact temperature value as in the case of pNiPAAm or linear PILs. This is due to the PIL polymer chains being crosslinked, with decreased freedom to shrink owing to the highly bulky and charged nature of the ionic liquid monomers [3,15].

**Figure 11:**
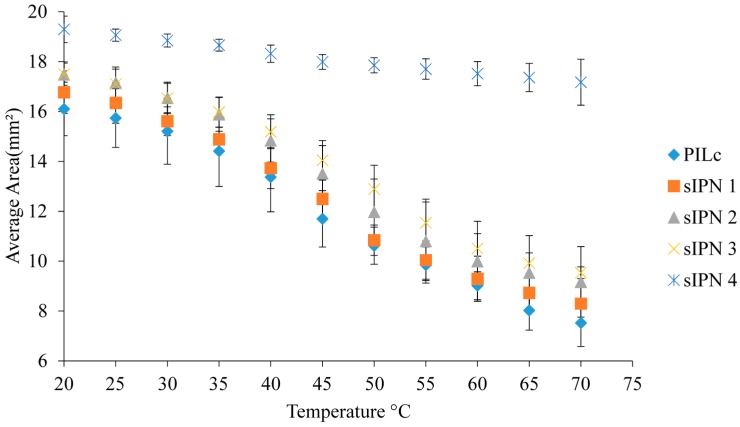
Graphical representation of the temperature induced shrinking profiles of the hydrogels.

**Figure 12:**
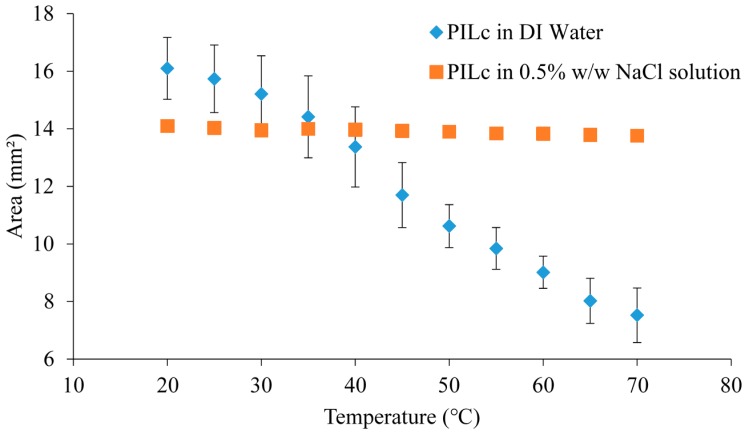
The shrinking profile of the PILc hydrogel in DI water and 0.5% w/w NaCl solution.

As described in the Experimental Section, the hydrogels were initially allowed to swell in DI water. For each measurement, three different hydrogels were used, to ensure that the process is reproducible. In Figure 11, the area difference at each temperature step is plotted against temperature to determine the influence of the linear p(NiPAAm-BSP-AA) copolymer on the temperature induced shrinking properties of the sIPN hydrogels. Using Equation (1), the area difference between the hydrogels at 20 °C and 70 °C was calculated (Table 5). PILc showed the highest degree of area shrinking at ~53.3% of its fully hydrated size, followed by sIPN 1, sIPN 2, and sIPN 3, with ~50.6%, ~47.6%, and ~45.5% area shrinking, respectively. These results indicate that the addition of the linear p(NiPAAm-BSP-AA) copolymer doesn’t significantly influence the thermally induced shrinking capabilities of the PIL matrix up to a linear copolymer:PILc molar ratio of 3:1. With sIPN 4 however, the shrinkage effect is much reduced at ~11%, suggesting that above a certain concentration of the copolymer, the linear chains inside the PILC matrix inhibit the collapse of the matrix. The effect of salt in the hydrating medium on the temperature response of the hydrogels was also investigated. In Figure 12, the temperature response of the PILc in the presence of 0.5% w/w NaCl was completely prevented. This result is consistent with a previous study done by Men *et al.* which showed that the LCST of different linear poly(ionic liquid)s is influenced by the presence of competing ions in the hydrating solution, to the point that the LCST completely disappears if the competing salt has a chaotropic behaviour [2]. In our experiments a 0.5% w/w NaCl concentration was enough to stabilize the PSPA chains in such a way that they did not collapse at any temperature. Furthermore, the 0.5% w/w NaCl solution had a similar effect with all the sIPNs, completely suppressing the temperature induced swelling in every case. 

**Table 5:** Temperature induced shrinking of the sIPNs in DI water.

Sample	Fully Swollen Hydrogel Area (mm^2^)	Standard Deviation (n = 3)	Contracted Hydrogel Area (mm^2^)	Standard Deviation (n = 3)	% Shrinking
**PILc**	16.101	1.074	7.524	0.945	53.273
**sIPN 1**	16.774	0.853	8.295	0765	50.549
**sIPN 2**	17.483	0.507	9.172	1.414	47.537
**sIPN 3**	17.497	0.443	9.537	0.232	45.494
**sIPN 4**	19.296	0.530	17.174	0.922	10.995

## 4. Conclusions

A series of multi-responsive crosslinked sIPN hydrogels were synthesised by adding increasing amounts of linear p(NiPAAm-BSP-AA) copolymer to a crosslinked PIL matrix. UV-Vis spectroscopy and DSC were used to determine how temperature and white light affect the linear copolymer. From the DSC data, it was determined that the LCST of the linear p(NiPAAm-BSP-AA) copolymer was at *ca.* 32.33 °C, while from the UV-Vis data it was found that white light irradiation promotes the precipitation of the linear copolymer from an aqueous solution at a lower temperature compared to the non-irradiated solution. Digital microscopy was used to determine the influence of white light, temperature, and salt concentration on the shrinking capabilities of sIPN hydrogels. From the gathered rheometry data, all hydrogels polymerized approximately after the same time period of 120 s, but have different mechanical properties that are influenced by the amount of linear p(NiPAAm-BSP-AA) copolymer used in their synthesis. The increasing amount of linear p(NiPAAm-BSP-AA) copolymer lowers the storage modulus and increases the loss modulus, thus the sIPNs become more tackier compared to the PILc. By analysing the shrinking response of the hydrogels after being exposed to photo-, thermo-, and salt stimuli, it was determined that sIPN 2 and sIPN 3 would be best suited to consider for use as polymer actuators (*e.g.*, in microfluidic systems), because they can be efficiently actuated by white light irradiation, salt concentration and an increase in temperature. Furthermore, this study shows that PILs can provide functional building blocks for the synthesis of novel smart materials, and provides an experimental template for the examination of other PILs as temperature and ionic strength responsive polymers, either by themselves or as components in composite materials. 
